# Metabolic reprogramming in bone metastasis of human cancers

**DOI:** 10.3389/fcell.2025.1711592

**Published:** 2025-12-18

**Authors:** Tianda Li, Ziyi Wang, Haitao Yang, Yihan Kang

**Affiliations:** 1 Department of Orthopedics, 242 Hospital Affiliated to Shenyang Medical College, Shenyang, Liaoning, China; 2 Department of Thoracic Surgery, National Cancer Center/National Clinical Research Center for Cancer/Cancer Hospital, Chinese Academy of Medical Sciences and Peking Union Medical College, Beijing, China; 3 Department of Thoracic Surgery, The People’s Hospital of Liaoning Province, Shenyang, China; 4 Department of Anesthesiology, The First Affiliated Hospital of China Medical University, Shenyang, Liaoning, China

**Keywords:** metabolic reprogramming, bone metastasis, hypoxia, microenvironment, glycolysis

## Abstract

Bone metastasis represents a complex complication of advanced human malignancies. Metabolic reprogramming plays a critical role in bone metastasis. Tumor cells hijack and alter local metabolic pathways to fuel their energetic and biosynthetic demands for proliferation and survival within the bone metastatic microenvironment. This includes adaptations in glycolysis, oxidative phosphorylation, lipid metabolism and amino acid metabolism. Furthermore, this bone metastatic microenvironment exhibits distinct metabolic features, such as hypoxia and acidity. To survive in this hostile microenvironment, tumor cells that metastasize to bone have to undergo metabolic reprogramming. Collectively, understanding the intricate link between metabolic reprogramming and bone metastasis is crucial for developing novel therapeutic strategies. Targeting the specific metabolic addiction and interrupting the nutrient-based crosstalk between tumor cells and the bone stroma offers a promising way to inhibit the vicious cycle and bone metastatic progression.

## Introduction

Bone is among the most frequent sites of distant metastasis in advanced cancer (Yin et al., 2005). Bone metastasis happens in 70% of patients with metastatic breast or prostate cancer, and 15%–30% of those with lung, renal, or thyroid carcinoma (Fornetti et al., 2018; Hu et al., 2023). Bone metastasis not only contributes to the disease burden by causing skeletal-related events such as pain, pathological fractures, spinal cord compression, and hypercalcemia, but also significantly shortens overall survival of patients (Yang et al., 2019). In recent years, the treatment of metastatic bone disease has witnessed remarkable progress with the emergence of novel therapeutic approaches, such as ^177^Lu-labeled PSMA-targeted radioligand-therapy for prostate cancer and antibody-drug conjugates for breast cancer (Kratochwil et al., 2023; Tian et al., 2022). These approaches not only transcend the limitations of conventional supportive care but also act precisely on tumor lesions within the bone microenvironment, bringing about substantial transformation in the clinical management of metastatic bone disease. Nevertheless, the development of therapeutic strategies that can precisely target bone metastases remains of critical importance.

Metabolic reprogramming plays a critical role in cancer metastasis (Faubert et al., 2020). Firstly, it supplies tumors with energy and biomolecules by altering metabolic pathways, thereby supporting their participation in pre-metastatic niche formation and colonization. Secondly, it enables tumor cells to adapt to harsh tumor microenvironment (TME) conditions, such as hypoxia and acidosis, thereby sustaining survival, proliferation, and creating a favorable ecological niche for metastasis. Additionally, metabolic reprogramming modulates intrinsic signaling pathways that regulate cellular proliferation, migration, and invasion, further promoting tumor growth and dissemination (Altea-Manzano et al., 2025). During migration and colonization to bone tissue, tumor cells must reprogram their metabolic states to accommodate the bone marrow niche, which possesses distinct nutritional and microenvironmental features. The bone marrow microenvironment is metabolically unique, and cancer stem cells also reprogram their metabolism not only to survive but to thrive in this niche. The metabolic adaptation is a prerequisite for every step of the bone metastatic cascade (Stouras et al., 2023). Recent studies have revealed that tumor cells promote bone metastasis through adaptations in glucose, lipid, and amino acid metabolism. However, the underlying mechanisms, functional impact, and translational potential of metabolic reprogramming in bone metastasis warrant further investigation.

## Tumor cell adaptation to bone metastatic microenvironments

The bone microenvironment provides a fertile niche that facilitates the homing, proliferation, and survival of various malignancies (Hofbauer et al., 2021). As a dynamic organ, bone consists of multiple specialized cell populations from diverse embryonic origins, including hematopoietic, stromal, and endothelial lineages, that together establish a distinctive niche for metastasis (Morrison and Scadden, 2014; Emoto et al., 2022). Two key cell types, osteoclasts and osteoblasts, jointly regulate bone remodeling during bone development (Florencio-Silva et al., 2015). Osteoclasts, derived from mononuclear-phagocyte lineage precursors, are responsible for bone resorption, whereas osteoblasts, originating from mesenchymal stromal progenitors, mediate new bone matrix deposition (Florencio-Silva et al., 2015). This bone metastatic microenvironment exhibits distinct metabolic features, such as hypoxia and acidity. To survive in this hostile microenvironment, tumor cells that metastasize to bone have to undergo metabolic reprogramming.

### Adaptation to hypoxic bone metastatic microenvironments

Hypoxia is a well-established hallmark of solid tumors (Jing et al., 2019). Rapid proliferation of malignant cells leads to metabolic demands that exceed the oxygen supply capacity of the existing vasculature. This oxygen deprivation acts as a critical driver of tumor adaptation, promoting angiogenic responses and activating cellular survival pathways. The HIF signaling pathway plays a central role by regulating genes involved in angiogenesis and metabolism, facilitating cellular adaptation to low oxygen conditions and stimulating angiogenesis to restore oxygen homeostasis. Clinically, higher HIF1α expression and activation of HIF signaling pathway has been observed in primary breast tumor samples from patients with bone metastasis compared to those without bone metastasis (Woelfle et al., 2003; Todd and Johnson, 2020).

The bone itself is a generally hypoxic organ, fueling the activation of HIF signaling in bone resident cells, promoting tumor cell homing to the bone as well as osteoclastogenesis. The hypoxic bone microenvironment further drives the vicious cycle of tumor-induced bone destruction, accelerating both tumor growth and osteolysis. Mitophagy, a selective type of macroautophagy, represents the mechanism for eliminating damaged or dysfunctional mitochondria under hypoxia. Deficiency in mitophagy due to ULK1 knockout enhances breast cancer bone metastasis by elevating ROS levels and activating the NLRP3 inflammasome. Notably, MAPK1/3 kinase facilitates BTRC-mediated proteasomal degradation of ULK1, and pharmacological inhibition of MAPK1/3 activation reduces bone metastasis and improves survival in mouse models (Deng et al., 2021).

### Adaptation to the extracellular acidification of bone metastatic microenvironments

**FIGURE 1:**
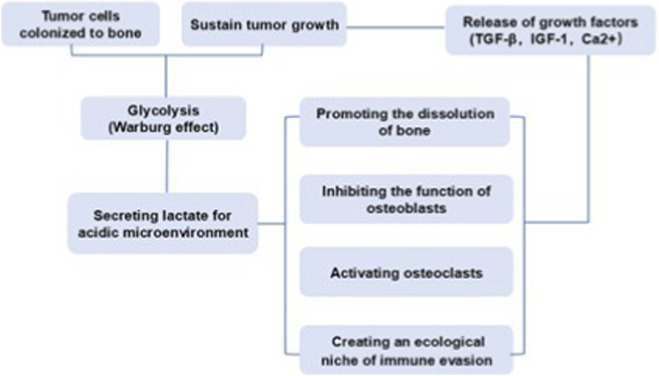
The bidirectional vicious cycle between acidic microenvironment and bone metastasis.

The high glycolysis rate of tumor cells leads to the secretion of a large amount of lactate, acidifying the local microenvironment (Doherty and Cleveland, 2013). The acidic environment not only directly promotes the dissolution of bone matrix, but also inhibits the function of osteoblasts, activates osteoclasts, and suppresses the activity of immune cells, creating a favorable ecological niche of “immune exemption” for tumor cells (Avnet et al., 2019). Moreover, the activation of osteoclasts leading to dissolution of bone matrix further promotes the release of various factors (such as TGF-β, IGF-1, Ca^2+^) stored in the bone matrix. These growth factors, in turn, further stimulate the proliferation of tumor cells and enhance their ability to secrete lactic acid, leading to a more acidic environment and more severe bone destruction. This bidirectional vicious cycle between acidic microenvironment and bone metastasis facilitates the colonization and growth of tumor cells in the bone metastatic foci (Figure 1). In simple terms, the acidic microenvironment is not only the result of bone metastasis of tumors but also an important cause driving its further deterioration.

Lactate plays an important role in osteoclast differentiation and function (Tiedemann et al., 2020). Thus, the metabolic reprogramming of breast cancer cells may contribute to osteolysis through lactate-mediated mechanisms. MCT1 and MCT4 are two of the major proton-coupled lactate symporters mediating bidirectional lactate transport across the plasma membrane (Sun et al., 2020). Many cancer cells express MCT1 and MCT4 through which substantial amounts of lactate resulting from Warburg effect are released, therefore MCTs control lactate signaling to create a favorable ecological niche for tumor cells to colonize in the bone (Qian et al., 2021). During osteolytic bone metastases, lactate released from tumor cells is up-taken and metabolically used by osteoclasts, the key players of osteolysis associated with bone metastasis. Specifically, MCT4 has been found to mediate the release of lactate from tumor cells at the bone site, while lactate is up-taken by osteoclasts via MCT1 to be used as a fuel for the oxidative metabolism of osteoclasts, ultimately enhancing Type I collagen resorption and facilitating bone resorption (Lemma et al., 2017). These metabolic alterations present a therapeutic opportunity for pharmacological intervention, as MCT1 inhibition disrupts the stimulatory effect of lactate on osteoclast activation. In addition, extracellular acidosis also suppresses the activity of immune cells in the bone microenvironment. For instance, Treg cells actively absorbed lactate through MCT1, promoting NFAT1 translocation into the nucleus, thereby enhancing the expression of PD-1 and creating an ecological niche of immune evasion (Kumagai et al., 2022).

## Therapeutic targeting key metabolic pathways involved in bone metastasis

**FIGURE 2:**
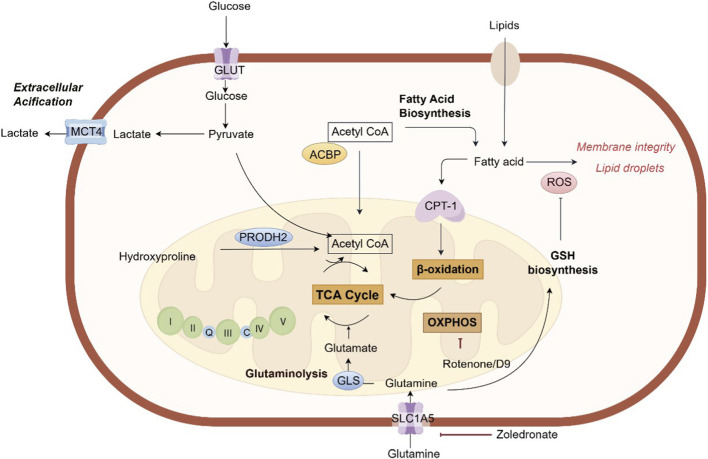
The key metabolic pathways involved in bone metastasis. ACBP, acyl-CoA-binding protein; CPT-1, carnitine palmitoyltransferase-1; GSH, glutathione; GLS, glutaminase; GLUT, glucose transporter; MCT4, monocarboxylate transporter 4; PRODH2, proline dehydrogenase 2; ROS, reactive oxygen species; SLC5A1, Na (+)/glucose cotransporter; TCA, tricarboxylic acid.

The successful establishment of bone metastasis from tumors not only depends on the metabolic reprogramming of tumor cells, but also on their “hijacking” of the metabolic activities of host bone cells (especially osteoclasts), jointly shaping a bone metastatic microenvironment that supports tumor growth, immunosuppression and bone destruction (Kfoury et al., 2021; Jackett et al., 2024). Thus, targeting the key metabolic nodes in this process has become a highly promising new strategy for the treatment of bone metastasis (Figure 2).

### Glycolysis

The existence of glycolysis in cancer cells has been observed *in situ* in patients with bone metastasis, as visualized by 18F-FDG-PET scanning of breast cancer bone metastases. Gene expression signatures revealed that breast cancer cells that preferentially metastasize to bone were more glycolytic, and consume less ATP and NADPH than parent MDA-MB-231 cells (Jekabsons et al., 2023). It has been found that pharmacologic blockade of LDHA, the rate-limiting enzyme of glycolysis, could mitigate prostate cancer development in bone (Kim et al., 2025). However, no LDHA-specific inhibitor is currently approved. While LDHA is a compelling target, developing specific and potent inhibitors with good pharmacokinetic properties remains a challenge. 2-Deoxy-D-glucose (2-DG), a glucose analog, inhibits hexokinase, the first and rate-limiting enzyme of glycolysis. While clinical trials have shown limited efficacy as a monotherapy, its use in combination with other agents is being explored. It has been found that a newly developed bone-targeted nano-platform combining with 2-DG significantly enhanced photodynamic therapy to inhibit bone metastasis (Li et al., 2024). Moreover, recent study has revealed that breast cancer fostered a hyperglycemic bone pre-metastatic niche before migrating to bone tissue and further enhances glucose metabolism following metastatic colonization (Ye et al., 2025). Subsequently, an intervention strategy has been proposed that targets glucose metabolism using a biomimetic-engineered enzyme-based nanoplatform to block bone metastasis of breast cancer. This platform employs membrane shielding to minimize interactions between the engineered glucose oxidase and circulating glucose for targeting of glucose metabolism. Herein, targeting glycolysis may be exploited as a promising strategy for the treatment of bone metastasis (Ye et al., 2025).

### Mitochondrial and oxidative phosphorylation

Mitochondria are dynamic intracellular organelles that sustain the biosynthetic and energetic demands of cancer cells, playing a central role in cellular metabolism through ATP production via the oxidative phosphorylation system (OXPHOS) (Vasan et al., 2020). Gene expression profiling has identified distinct metabolic programs in breast tumor cells that influence their metastatic patterns. OXPHOS was associated with metastasis to the lung and bone, whereas glycolytic reprogramming was correlated with liver metastasis (Dupuy et al., 2015).

Research has revealed that accumulation of calcified granules, resulting from breast cancer necrosis or spontaneous secretory deposits around mammary glands, induces reprogramming of tumor metabolism, with a notable increase in mitochondrial metabolic activity (Hu et al., 2020). This process is driven by upregulation of the OXPHOS complex and subsequent activation of TGFβ-SMAD and MAPK signaling pathways, which promote epithelial-mesenchymal transition (EMT) and facilitate bone metastasis of breast cancer cells. Intriguingly, the OXPHOS inhibitor rotenone was shown to suppress calcification-induced EMT, indicating that OXPHOS acts as a critical mechanistic link between calcification and EMT, and that its inhibition may help restrict metastatic progression. Specifically, OXPHOS blockade markedly reduced activation of TGFβ and MAPK pathways and reversed EMT phenotypes in breast cancer cells undergoing osteogenic differentiation (Hu et al., 2020). Through single-cell metabolic imaging via NADH fluorescence lifetime (FLIM) and analysis of Akt and ERK kinase activities, it was further demonstrated that breast cancer cells heavily rely on OXPHOS and Akt signaling within both 3D co-culture systems and murine models of ER + breast cancer metastasis in bone marrow (Buschhaus et al., 2020). Combinatorial treatment targeting OXPHOS with the thioredoxin reductase (TrxR) inhibitor D9 and the Akt inhibitor MK-2206 selectively eradicated breast cancer cells without affecting bone marrow stromal cells. *In vivo* experiments using mice with disseminated ER + human breast cancer showed that the combination of D9 and MK-2206 more effectively prevented the formation of new metastases compared to tamoxifen (Buschhaus et al., 2020).

### Lipid metabolism

Given the critical involvement of lipid metabolism in tumor cell survival, proliferation, and drug resistance, elucidating how tumor cells exploit lipid metabolic pathways to adapt to the bone microenvironment is essential for identifying novel therapeutic targets and developing effective interventions against bone metastasis (Currie et al., 2013; Broadfield et al., 2021). An *in vivo* CRISPR activation screen has identified acyl-CoA-binding protein (ACBP) as a key driver of bone metastasis (Teng et al., 2025). ACBP promotes cancer progression in bone by enhancing fatty acid oxidation (FAO). It binds to medium- and long-chain acyl-CoA molecules and facilitates their transport into mitochondria, thereby boosting energy production for tumor cells. Additionally, ACBP confers increased resistance to lipid peroxidation and ferroptosis, further supporting metastatic survival and growth in the bone microenvironment. A key challenge in bone metastasis is the persistence of dormant, treatment-resistant cells. These dormant disseminated tumor cells (DTCs) often shift their metabolism towards FAO mediated by the enzyme carnitine palmitoyl-transferase 1A (CPT-1A). This metabolic state allows them to survive for years in a quiescent state before being reactivated to form overt metastases. In mouse models of breast cancer and lung cancer, pharmacological inhibition of CPT1A, the rate-limiting enzyme in FAO, by etomoxir significantly suppressed bone metastasis (Teng et al., 2025). Collectively, targeting FAO represent a promising therapeutic approach for treating bone metastases.

Bone marrow adipocytes (BMAs), as major constituents of the bone marrow cavity, play a key role in regulating lipid metabolism within the bone microenvironment. BMAs serve not only as an energy reservoir for the bone niche but also function as an endocrine organ by secreting fatty acids, cytokines, and adipokines that facilitate cancer cell colonization and growth in the bone marrow. Emerging research on the metabolic functions of BMAs is critical for understanding how tumor cells successfully colonize and proliferate in this milieu. Studies have shown that BMAs supply energy directly to tumor cells through lipolysis and lipid transfer. Clinical evidence from bone metastasis specimens indicates lipid uptake in prostate cancer cells adjacent to BMAs, directly supporting their role in promoting tumor growth (Gazi et al., 2007). Moreover, BMAs remodel tumor cell metabolism to support proliferation and metastatic advancement. Specifically, BMA-derived lipids upregulate key lipid-handling proteins, such as CD36, FABP4, and Perilipin 2, thereby enhancing fatty acid uptake and utilization to improve prostate cancer cell survival (Herroon et al., 2013).

### Amino acid metabolism

Amino acids not only function as fundamental building blocks for proteins but also act as key signaling molecules that regulate essential biological processes (Sivanand and Vander Heiden, 2020). Growing evidence highlights the central role of metabolic reprogramming, particularly of amino acid metabolism, within the bone metastatic microenvironment (Bergers and Fendt, 2021). These metabolic alterations significantly influence critical stages of metastasis, such as tumor dormancy, colonization, and reactivation. Consequently, the reprogramming of amino acid metabolism represents a promising therapeutic target for disrupting bone metastasis.

Glutamine, a nonessential amino acid, is the most abundant amino acid in the bloodstream. Metastatic tumor cells upregulate glutaminase (GLS), breaking down the abundant amino acid glutamine to fuel the TCA cycle and generate antioxidants (Jin et al., 2023). This “glutamine addiction” is critical for the adaptation of metastatic tumor cells in the bone metastatic microenvironment characterized by high oxidative stress. Consequently, tumor cells depend on glutamine metabolism to support their colonization within bone tissue (Bergers and Fendt, 2021). In breast cancer, osteoclasts facilitate the survival of tumor cells against DNA-damaging agents by releasing glutamine (Fan et al., 2024). Cancer cells efficiently take up glutamine via the transporter SLC1A5 and metabolize it through GLS1 to mitigate DNA damage. Mechanistically, glutamine enhances GSH synthesis in tumor cells, which neutralizes excessive ROS generated during DNA damage, thereby reducing cellular damage and promoting cell survival within the bone metastatic microenvironment. A combination therapy approach using PARP inhibitors (PARPi) and zoledronate, which suppresses osteoclast activity and limits glutamine availability, demonstrated synergistic efficacy in reducing bone metastasis (Fan et al., 2024).

Hydroxyproline is a key amino acid produced from collagen degradation, providing stability to the collagen structure (Srivastava et al., 2016). Recent studies have revealed that it functions as an active metabolic signaling molecule influencing multiple aspects of tumor progression (Chalecka et al., 2025). During bone metastasis, aberrant osteoclast activation leads to bone matrix degradation. Clinically, elevated hydroxyproline levels in serum and urine are highly correlated with the occurrence of bone metastasis and are regarded as potential biomarkers for bone metastasis of breast cancer (Gielen et al., 1976). Hydroxyproline can be catabolized by the metabolic enzyme proline dehydrogenase 2 (PRODH2) and enter the TCA cycle, generating energy and biosynthetic precursors to fuel rapidly proliferating tumor cells (Summitt et al., 2015; Buchalski et al., 2020). As the core enzyme in hydroxyproline metabolism, PRODH2 was significantly upregulated in clinical samples from BC bone metastases (Gong et al., 2025). Notably, PRODH2-mediated hydroxyproline metabolism was found to promote osteoclast differentiation, which enhanced collagen degradation and facilitated breast cancer bone metastasis *in vivo*. Furthermore, PRODH2 supported tumor cell survival by upregulating the ferroptosis inhibitor SLC7A11, and stimulated osteoclast differentiation through increasing the production of the metastasis-related cytokine IL-8. Mechanistically, PRODH2-driven hydroxyproline metabolism generated acetyl-CoA, which enhanced YY1 acetylation, leading to transcriptional activation of both SLC7A11 and IL-8. N-propargylglycine, the PRODH2 inhibitor, significantly reduced osteoclast differentiation *in vitro* and inhibited bone metastasis, osteoclast differentiation, and collagen degradation *in vivo*. Collectively, these findings demonstrate that collagen breakdown during osteolysis releases hydroxyproline, which in turn fuels a vicious cycle by further driving osteoclast differentiation and metastatic progression (Gong et al., 2025).

Serine metabolism plays a pivotal role in cancer and has emerged as an attractive therapeutic target. In tumor cells, approximately 10% of the glycolytic intermediate 3-phosphoglycerate is oxidized into the serine precursor 3-phosphohydroxypyruvate in a reaction catalyzed by phosphoglycerate dehydrogenase (PHGDH) using NAD^+^ as a cofactor. This product is then converted to serine through sequential enzymatic reactions: transamination by phosphoserine aminotransferase (PSAT1) and hydrolysis by phosphoserine phosphatase (PSPH). Notably, serine promotes the differentiation of human osteoclasts capable of bone resorption, suggesting a contribution to the vicious cycle of osteolytic bone metastasis. Consistent with this, PHGDH, PSAT1, and PSPH were significantly upregulated in highly bone-metastatic MDA-MB-231 cells. The stimulatory effects of L-serine on both osteoclastogenesis and cancer cell proliferation underscore the functional importance of serine biosynthesis in bone-metastatic breast cancer, highlighting a promising avenue for targeted therapy (Pollari et al., 2011). It has been found that PHGDH-amplified cells are highly dependent on this enzyme for bone metastasis. Genetic or pharmacological inhibition of PHGDH by NCT-503 preferentially impaired the growth of PHGDH-amplified breast cancer cells in bone marrow-mimicking conditions and in intra-tibial xenograft models by inducing oxidative stress and nucleotide depletion (Possemato et al., 2011). However, no PHGDH inhibitor has reached late-stage clinical trials yet.

## Conclusion

In this review, we underscore the pivotal role of tumor metabolism in the establishment and progression of bone metastases. Metabolic adaptations, scavenging nutrient from the bone matrix, empower cancer cells to survive, proliferate, and thrive in the hypoxic and acidic bone marrow. Understanding this intricate interplay provides a strong rationale for developing novel therapeutic strategies that simultaneously target the metabolic vulnerabilities of tumor cells and disrupt the signaling loops within the bone microenvironment. Future directions should focus on combining conventional bone-targeting agents with novel drugs that disrupt critical metabolic pathways in tumor cells. Additionally, targeting the metabolic crosstalk itself offers a compelling approach to break the vicious cycle, potentially leading to more effective and targeted treatments for patients with bone metastasis.
